# Plant Spatio-Temporal Integration Network (PSTN): a framework for dynamic single-cell and spatial transcriptomics in fungal–plant interactions

**DOI:** 10.1093/bioinformatics/btag637

**Published:** 2026-08-22

**Authors:** Yuheng Zhu, Hao Zhang, Xianyu Zhang, Enshuang Zhao, Yinfei Dai, Hanbo Liu, Longyi Li, Xiyuan Mei, Yuxin Jin

**Affiliations:** College of Computer Science and Technology, Jilin University, Jilin 130012, China; College of Computer Science and Technology, Jilin University, Jilin 130012, China; College of Plant Protection, China Agricultural University, Beijing 100193, China; College of Computer Science and Technology, Jilin University, Jilin 130012, China; College of Computer Science and Technology, Jilin University, Jilin 130012, China; College of Computer Science and Technology, Jilin University, Jilin 130012, China; College of Computer Science and Technology, Jilin University, Jilin 130012, China; College of Computer Science and Technology, Jilin University, Jilin 130012, China; College of Computer Science and Technology, Jilin University, Jilin 130012, China

## Abstract

**Motivation:**

Rice blast caused by Magnaporthe oryzae (*M. oryzae*) is a dynamic cross-kingdom infection process involving rapid transcriptional reprogramming and spatial tissue remodeling across successive stages. Although single-cell RNA sequencing (scRNA-seq) and spatial transcriptomics (ST) provide complementary information, most integration methods analyze stages independently and ignore temporal continuity.

**Results:**

We developed the Plant Spatio-Temporal Integration Network (PSTN), which jointly learns stage-specific cell-to-space mappings through expression reconstruction and bidirectional maximum-similarity temporal regularization. Applied to matched rice scRNA-seq and ST data at 0, 12, and 24 h, PSTN outperformed Tangram, SpaGE, cell2location, and DestVI across three gene-set sizes. At 4000 highly variable genes (HVGs), mean squared error (MSE) remained below 0.09 at all stages, with high gene-wise and spot-wise correlations. Mapping-structure and marker-based analyses recovered tissue-associated spatial and temporal patterns. Gene-permutation controls markedly reduced performance, demonstrating reliance on correct cross-modal gene correspondence. An independent mouse-cortex benchmark supported the static reconstruction component across systems. PSTN enables temporally coupled single-cell and spatial integration in dynamic host–pathogen systems.

**Availability:**

PSTN is available at https://github.com/zhuyuheng111/PSTN. The Zenodo DOI for the repository is 10.5281/zenodo.21365720.

## Graphical Abstract

**Graphical Abstract:**
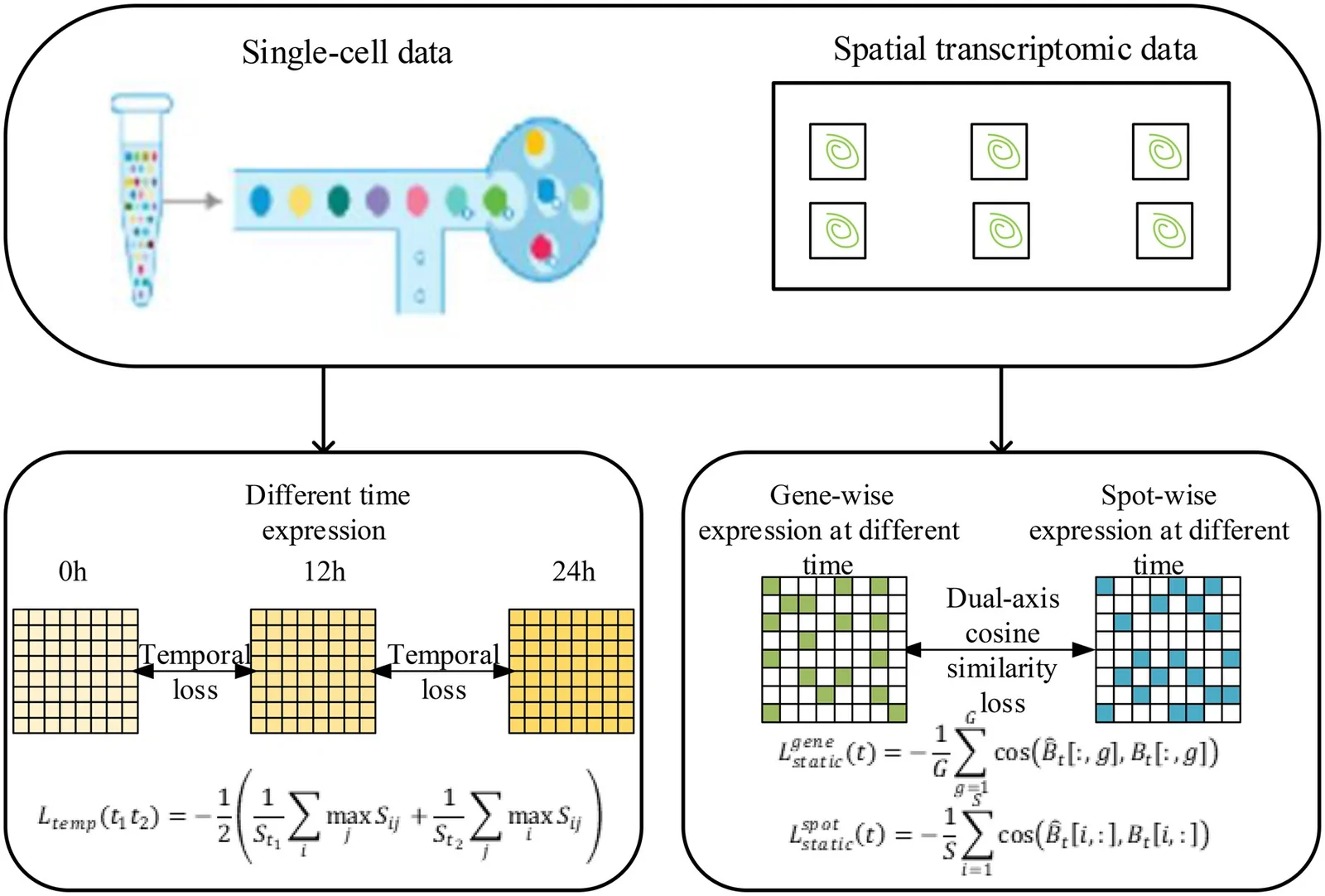
PSTN: Integrates single-cell and spatial data across infection stages.

## 1. Introduction

Rice blast, caused by *Magnaporthe oryzae* (*M. oryzae*), is a major threat to global rice production and can reduce yields by up to 30% (Nalley *et al.* 2016). Infection proceeds through appressorium formation, host penetration, biotrophic colonization, and necrotrophic spread, accompanied by coordinated molecular and tissue-level changes (Jeon *et al.* 2020, Wang *et al.* 2024, Wengler and Talbot 2025). These processes involve extensive transcriptional reprogramming and spatial reorganization of host tissues, making their spatio-temporal characterization important for understanding plant defense and improving disease resistance (Soanes *et al.* 2012, Eseola *et al.* 2021, Yan *et al.* 2023, Wang *et al.* 2024).

Single-cell RNA sequencing (scRNA-seq) and spatial transcriptomics (ST) provide complementary views of such dynamic processes (Tang *et al.* 2009, Hwang *et al.* 2018, Svensson *et al.* 2018, Zhou *et al.* 2023, Zhu *et al.* 2023). scRNA-seq resolves cellular heterogeneity, rare populations, and dynamic states that are obscured in bulk measurements (Zhu *et al.* 2023, Wang *et al.* 2025), whereas ST preserves tissue context and enables the identification of spatial domains, gradients, and intercellular niches (Ståhl *et al.* 2016, Cang and Nie 2020, Maynard *et al.* 2021, Rao *et al.* 2021). However, scRNA-seq loses spatial information during tissue dissociation (Adema *et al.* 2024), while spot-based ST platforms such as 10× Visium capture mixtures of multiple cells and therefore have limited cellular resolution (Ståhl *et al.* 2016, Cheng *et al.* 2023).

Several computational frameworks have been developed to integrate scRNA-seq and ST. Tangram learns cell-to-space mappings from shared expression profiles, whereas SpaGE focuses on spatial gene-expression prediction (Abdelaal *et al.* 2020, Biancalani *et al.* 2021). Stereoscope, RCTD, cell2location, and DestVI infer discrete cell-type mixtures or continuous cellular states within spatial locations (Andersson *et al.* 2020, Cable *et al.* 2022, Kleshchevnikov *et al.* 2022, Lopez *et al.* 2022). SPOTlight uses seeded non-negative matrix factorization and regression, while novoSpaRc reconstructs spatial organization through optimal transport (Nitzan *et al.* 2019, Elosua-Bayes *et al.* 2021). Despite their success, these methods are generally applied to individual spatial samples and do not explicitly couple mappings across successive biological stages. This limitation is particularly relevant to rice blast, in which host transcriptional programs and tissue organization change continuously during infection (Wilson and Talbot 2009, Fernandez *et al.* 2014, Fernandez and Orth 2018, Li *et al.* 2022).

Existing time-aware approaches, including RNA velocity methods such as scVelo and dynamic graph models such as T-GCN and EvolveGCN, primarily model temporal variation within a single modality and do not directly address joint scRNA-seq–ST integration across stages (Zhao *et al.* 2019, Bergen *et al.* 2020, Pareja *et al.* 2020). Here, we propose the Plant Spatio-Temporal Integration Network (PSTN), which extends established cell-to-space mapping principles to a jointly optimized multi-stage framework. PSTN combines stage-specific expression reconstruction with explicit temporal coupling across consecutive stages. Its main contributions are:

1. A joint optimization framework that simultaneously learns stage-specific cell-to-space mapping matrices, replacing independent snapshot-wise estimation with a unified spatio-temporal formulation;
2. A correspondence-free temporal regularization strategy based on bidirectional maximum-similarity alignment, allowing adjacent stages with different numbers of spatial spots to be coupled without predefined trajectories or one-to-one spot correspondence;
3. A comprehensive evaluation in the *M. oryzae*–rice infection system, complemented by an independent static benchmark on mouse-cortex scRNA-seq–Visium data, assessing reconstruction accuracy, tissue-associated spatial recovery, residual structure, histological concordance, and cross-system applicability.

## 2. Materials and methods

### 2.1 Overview of the PSTN framework

**Figure 1:**
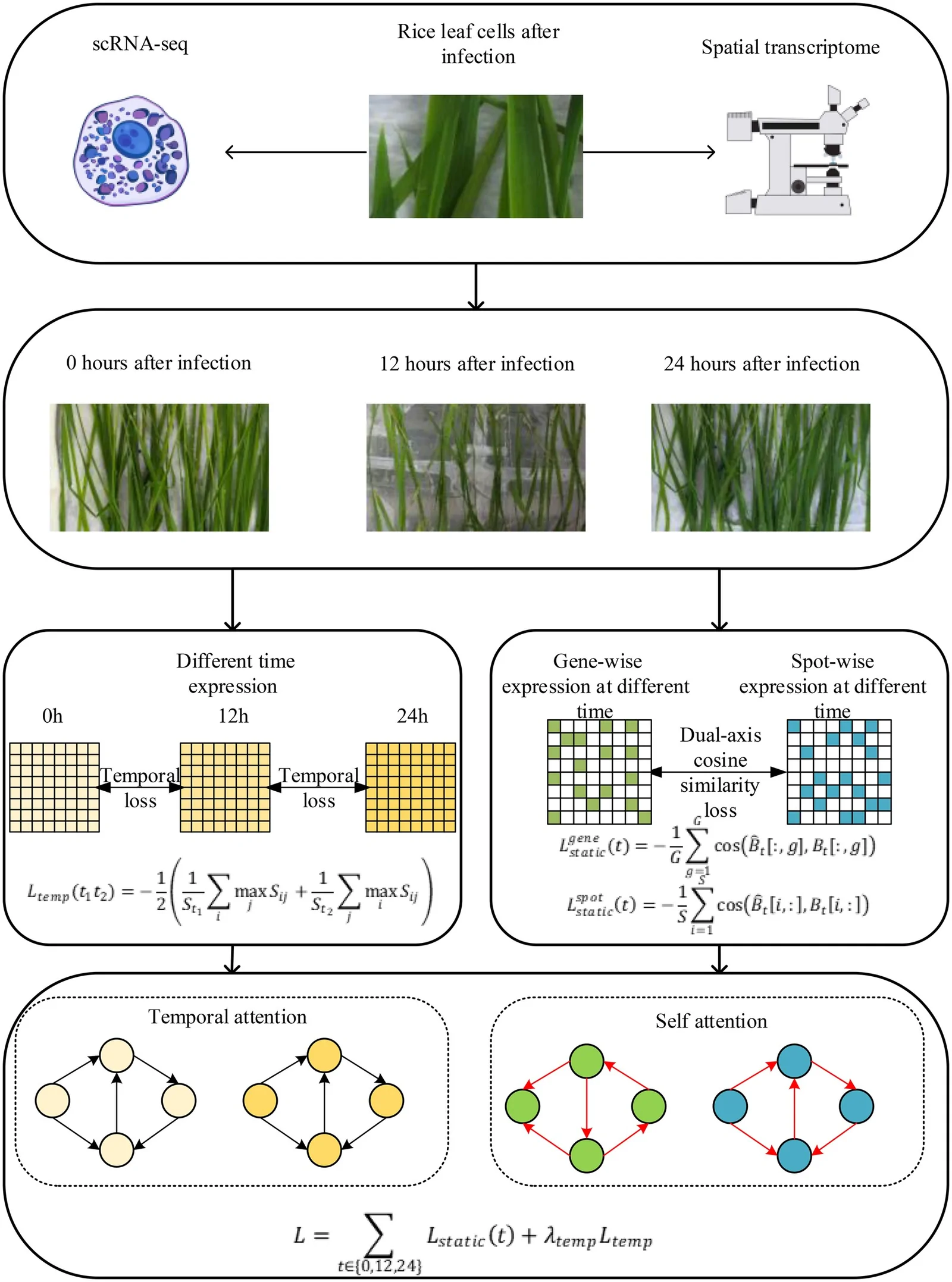
PSTN workflow. Rice leaf scRNA-seq and ST data were collected at 0, 12, and 24 h post *M. oryzae* infection. After quality control and marker-gene screening, the processed scRNA-seq and ST matrices were used as inputs to PSTN.

Figure 1 summarizes the PSTN workflow. After harmonizing scRNA-seq and ST data from 0 h, 12 h, and 24 h into shared highly variable genes (HVGs) panels, PSTN jointly optimizes stage-specific cell-to-space mapping matrices using static reconstruction and temporal-consistency losses.

### 2.2 Dataset and preprocessing

We loaded the raw h5ad files containing scRNA-seq and ST data at 0 h, 12 h, and 24 h post-infection (Wang *et al.* 2025). The scRNA-seq and ST datasets are available from the NCBI Sequence Read Archive under BioProject accession numbers PRJNA1121079 and PRJNA1150774. Details of these datasets are summarized in Table 1. Standard quality control was performed to remove cells or spots with low detected gene counts.

**Table 1:** Details of scRNA-seq and ST data.

Dataset	ST (0 h)	ST (12 h)	ST (24 h)	scRNA-seq (0 h)	scRNA-seq (12 h)	scRNA-seq (24 h)
Spots/cells	985	554	959	42 079	47 570	53 374
Genes	18 494	19 202	18 494	40 008	40 008	40 008

Raw count matrices used for model fitting were retrieved from AnnData.layers[“raw_X”] when available, or from AnnData.raw.X when a dimensionally compatible raw representation was present. In parallel, HVGs selection was performed in the library-size-normalized and log1p-transformed space after restricting the six datasets to the shared gene set and intersecting it with the curated rice marker gene set (Chen *et al.* 2021). Genes were ranked by variance across the six normalized log1p matrices, and the top 1000, 2000, and 4000 genes were selected as HVGs panels. For each panel, the raw-count and normalized representations were subset consistently for subsequent modeling.

Statistical analysis was performed on per-gene Pearson correlations using Wilcoxon signed-rank tests with Benjamini–Hochberg false discovery rate (FDR) correction; effect sizes were summarized as median differences with 95% bootstrap confidence intervals. Differences with FDR-adjusted P<0.01 were considered statistically significant.

### 2.3 Model formulation

PSTN combines stage-specific expression reconstruction with temporal coupling between successive stages. The complete training procedure is summarized in Algorithm 1.

Algorithm 1 Training procedure of PSTN

**Require**: Time points T={0,12,24}; adjacent-stage pairs P={(0,12),(12,24)}; single-cell raw-count matrices $\left\{A_{t}\right\}$; observed spatial expression matrices $\left\{B_{t}\right\}$; temporal regularization weight $\lambda_{\text{temp}}$; target library size $L=10^{4}$; numerical-stability constant ϵ; number of epochs *E*.

**Ensure**: Trained cell-to-spot mapping matrices $\left\{M_{t}\right\}$ and reconstructed spatial expression matrices $\left\{{\hat{B}}_{t}\right\}$.

1: Initialize trainable mapping matrices $\left\{M_{t}\right\}$ for all t∈T.

2: **for** epoch =1,…,E  **do** 

3:   **for** each stage t∈T  **do** 

4:    Reconstruct raw spatial expression: ${\hat{B}}_{t}^{\text{raw}}\leftarrow M_{t}^{⊤}A_{t}$.

5:    Clamp negative values in ${\hat{B}}_{t}^{\text{raw}}$ to zero.

6:    Normalize each spot to total counts *L* and apply the log1p transformation to obtain ${\hat{B}}_{t}$.

7:    Compute the gene-wise and spot-wise static reconstruction losses according to Equations (4)–(5).

8:    Set $L_{\text{static}}(t)\leftarrow L_{\text{static}}^{\text{gene}}(t)+L_{\text{static}}^{\text{spot}}(t)$.

9:    Row-normalize ${\hat{B}}_{t}$ to obtain the directional embedding matrix $E_{t}$.

10:   **end for** 

11:   Set $L_{\text{temp}}\leftarrow 0$.

12:   **for** each adjacent-stage pair $(t_{1},t_{2})\in P$  **do** 

13:    Compute cross-stage cosine similarity: $S_{t_{1},t_{2}}\leftarrow E_{t_{1}}E_{t_{2}}^{⊤}$.

14:    Compute the bidirectional maximum-similarity temporal loss according to Equation (9).

15:    Accumulate $L_{\text{temp}}$ over adjacent-stage pairs according to Equation (10).

16:   **end for** 

17:    Compute $L\leftarrow \sum_{t\in \{0,12,24\}}L_{\text{static}}(t)+\lambda_{\text{temp}}L_{\text{temp}}$.

18:    Update all $\left\{M_{t}\right\}$ jointly using Adam to minimize L.

19: **end for** 

20: **return**  $\left\{M_{t}\right\}$ and $\left\{{\hat{B}}_{t}\right\}$.

The training procedure of PSTN is summarized in Algorithm 1. All objectives are optimized under a unified evaluation space, where predicted spatial expressions are obtained by per-spot library-size normalization to $10^{4}$ followed by log1p transformation. The bidirectional cosine similarity is computed along both gene-wise and spot-wise dimensions, and temporal consistency is enforced via bidirectional maximum-similarity alignment between consecutive stages.

At each stage t∈{0,12,24}, let the single-cell expression matrix be $A_{t}\in R^{C\times G}$, and the spatial expression matrix be $B_{t}\in R^{S\times G}$, where *C*, *S*, and *G* denote the number of single cells, spatial spots, and HVGs, respectively. A trainable mapping matrix $M_{t}\in R^{C\times S}$ is introduced to project single-cell profiles onto spatial spots:


(1) $${\hat{B}}_{t}=M_{t}^{T}A_{t}$$

Here, $M_{t}\in R^{C\times S}$ denotes a cell-to-spot mapping matrix, where rows correspond to single cells and columns correspond to spatial spots. In the current implementation, $M_{t}$ is optimized as a free trainable matrix without imposing a row-wise or column-wise simplex constraint during training. Equation (1) represents each spatial spot as a trainable linear combination of single-cell expression profiles. This formulation provides a direct cell-to-space reconstruction operator while avoiding additional architectural complexity.

### 2.4 Cosine similarity and preprocessing

Losses are computed in the per-spot-normalized log1p space. Specifically, for each stage, the raw single-cell count matrix retrieved as described in Section 2.2 is first projected onto spatial coordinates as:


(2) $${\hat{B}}_{t}^{\text{raw}}=M_{t}^{⊤}A_{t},\;(t\in \{0,12,24\})$$

where ${\hat{B}}_{t}^{\text{raw}}$ denotes the reconstructed raw spatial expression matrix. Subsequently, the reconstructed matrix is clamped element-wise at zero, normalized to $10^{4}$ total counts per spot, and log1p-transformed to obtain the normalized prediction ${\hat{B}}_{t}$.

The supervision signal $B_{t}$ is generated using the same preprocessing protocol, including per-spot normalization and log(1+x) transformation. Cosine similarity is defined as:


(3) $$\text{cos}(u,v)=\frac{u\cdot v}{||u|{|}_{2}||v|{|}_{2}+\epsilon }$$

Cosine similarity was used because it is less sensitive than mean squared error (MSE) to scale variation across genes and spots. A small ϵ was included for numerical stability.

### 2.5 Static reconstruction loss

The static loss aligns predicted and observed expression along both gene and spot dimensions:

*Gene-wise alignment:*


(4) $$L_{\text{static}}^{\text{gene}}(t)=-\frac{1}{G}\sum_{g=1}^{G}\;\text{cos}\;\left({\hat{B}}_{t}[:,g],B_{t}[:,g]\right)$$

Equation (4) encourages alignment between predicted and observed expression profiles across genes, ensuring that the spatial patterns of individual genes are preserved.

*Spot-wise alignment:*


(5) $$L_{\text{static}}^{\text{spot}}(t)=-\frac{1}{S}\sum_{i=1}^{S}\;\text{cos}\;\left({\hat{B}}_{t}[i,:],B_{t}[i,:]\right)$$

Equation (5) aligns predictions with observations at the spot level, ensuring accurate reconstruction of each spot’s full transcriptome profile.

The combined static reconstruction loss is defined as:


(6) $$L_{\text{static}}(t)=L_{\text{static}}^{\text{gene}}(t)+L_{\text{static}}^{\text{spot}}(t)$$

Equal weights are assigned to the gene-wise and spot-wise loss terms to balance both constraints without introducing additional hyperparameters.

### 2.6 Temporal consistency loss

First, we row-normalize the predicted spatial expression matrix ${\hat{B}}_{t}$ to obtain per-spot directional embeddings:


(7) $$E_{t}[i,:]=\frac{{\hat{B}}_{t}[i,:]}{||{\hat{B}}_{t}[i,:]|{|}_{2}+\epsilon }$$

where $E_{t}\in R^{S\times G}$ denotes the normalized embedding matrix at stage *t*.

For consecutive stages $\left(t_{1},t_{2})\in \{(0,12),(12,24)\right\}$, we compute the cosine similarity matrix:


(8) $$S_{t_{1},t_{2}}=E_{t_{1}}E_{t_{2}}^{⊤}$$

where $S_{t_{1},t_{2}}\in R^{S_{t_{1}}\times S_{t_{2}}}$ encodes pairwise similarities between spatial spots at consecutive stages.

We then define a bidirectional maximum-similarity objective by averaging the row-wise and column-wise maxima of the cross-stage similarity matrix:


(9) $$L_{\text{temp}}(t_{1},t_{2})=-\frac{1}{2}\left(\frac{1}{S_{t_{1}}}\sum_{i}\underset{j}{\text{max}}S_{ij}+\frac{1}{S_{t_{2}}}\sum_{j}\underset{i}{\text{max}}S_{ij}\right)$$

where $S_{t_{1}}$ and $S_{t_{2}}$ denote the numbers of spatial spots at stages $t_{1}$ and $t_{2}$, respectively.

The total temporal regularization loss is defined as the sum over all adjacent stage pairs:


(10) $$L_{\text{temp}}=L_{\text{temp}}(0,12)+L_{\text{temp}}(12,24)$$

The final training objective is:


(11) $$L=\sum_{t\in \{0,12,24\}}L_{\text{static}}(t)+\lambda_{\text{temp}}L_{\text{temp}}$$

where $\lambda_{\text{temp}}$ controls the strength of the temporal regularization term. Unless otherwise specified, we treat $\lambda_{\text{temp}}$ as a tunable hyperparameter and examine its impact through sensitivity analysis. Based on this analysis, we set $\lambda_{\text{temp}}=0.5$ as the default value in all experiments.

The clamp operation is applied to the reconstructed expression $M_{t}^{⊤}A_{t}$, not to $M_{t}$. Negative reconstructed values are set to zero before normalization and log1p transformation. All stage-specific mapping matrices are optimized jointly according to Equation (11).

### 2.7 Gene-permutation negative-control analysis

To assess whether reconstruction depended on valid cross-modal gene correspondence, we performed a gene-permutation negative-control analysis using the 4000-HVGs matrices. The scRNA-seq gene columns were randomly deranged using the same permutation across 0 h, 12 h, and 24 h, while the ST matrices remained unchanged. PSTN and Tangram were then retrained under the same settings used for their corresponding real-input analyses and evaluated using MSE, mean gene-wise Pearson correlation, and mean spot-wise Pearson correlation. Detailed results are provided in Table S3, available as supplementary data at *Bioinformatics* online.

## 3. Experiments and results

### 3.1 Evaluation metrics

All metrics are computed in the per-spot-normalized log1p space, consistent with training. For stage *t* with $S_{t}$ spots and *G* genes, let ${\hat{B}}_{t},B_{t}\in R^{S_{t}\times G}$ denote predicted and observed matrices.

*Global MSE:* The overall element-wise reconstruction error is measured by the MSE:


(12) $$\mathrm{MSE}(t)=\frac{1}{S_{t}G}\sum_{i=1}^{S_{t}}\sum_{g=1}^{G}{\left({\hat{B}}_{t}[i,g]-B_{t}[i,g]\right)}^{2}$$

*Gene-wise Pearson correlation:* For each gene *g*, compute Pearson’s correlation coefficient across spatial spots and then average over all genes:


(13) $$r_{g}^{\text{gene}}(t)=\text{corr}\left({\hat{B}}_{t}[:,g],B_{t}[:,g]\right)$$

(14) $${\bar{R}}^{\text{gene}}(t)=\frac{1}{G}\sum_{g=1}^{G}r_{g}^{\text{gene}}(t)$$

*Spot-wise Pearson correlation:* Similarly, for each spatial spot *i*, Pearson’s correlation coefficient is computed across genes and then averaged over all spots:


(15) $$r_{i}^{\text{spot}}(t)=\text{corr}\left({\hat{B}}_{t}[i,:],B_{t}[i,:]\right)$$

(16) $${\bar{R}}^{\text{spot}}(t)=\frac{1}{S_{t}}\sum_{i=1}^{S_{t}}r_{i}^{\text{spot}}(t)$$

These metrics are used in prior mapping studies, enabling direct comparison with Tangram, SpaGE, cell2location, and DestVI.

### 3.2 Implementation details

All experiments were conducted on a GPU workstation equipped with an NVIDIA RTX 3090 GPU (24 GB video memory). PSTN was implemented in Python 3.9 and optimized for 2000 epochs using Adam with a learning rate of $1\times 10^{-3}$. The temporal regularization weight, $\lambda_{\text{temp}}$, was selected through sensitivity analysis, and all stage-specific mapping matrices were jointly optimized within a single training run.

For the rice infection benchmark, Tangram, SpaGE baseline, cell2location, and DestVI were applied independently at 0 h, 12 h, and 24 h using the corresponding 1000, 2000, and 4000 HVGs panels. Detailed software information, input representations, gene-set handling, key hyperparameters, and output-conversion procedures are provided in Supplementary Note S2, available as supplementary data at *Bioinformatics* online. All reconstructed spot-by-gene matrices were aligned to the same gene order and evaluated using an identical pipeline, including non-negative clipping when applicable, per-spot normalization to $10^{4}$ total counts, log1p transformation, global MSE, mean gene-wise Pearson correlation, and mean spot-wise Pearson correlation.

For the independent mouse-cortex benchmark, we used 21 697 annotated cortical cells, 324 spatial spots, and 1382 shared marker genes derived from the union of the top 100 markers for each cell subclass and retained in both modalities. PSTN was applied with temporal regularization disabled, whereas Tangram was run in cell-level mapping mode with an RNA-count-based density prior for 2000 epochs. Both methods were repeated using three independent random initializations and evaluated using common spot-wise and gene-wise cosine-similarity metrics.

### 3.3 Temporal-weight sensitivity and overall reconstruction performance

We evaluated the sensitivity of PSTN to the temporal weight $\lambda_{\text{temp}}$ (HVGs = 4000; Table 2). Overall reconstruction performance improved as $\lambda_{\text{temp}}$ increased to 0.5, although individual metrics did not change monotonically across all stages. Values larger than 0.5 substantially increased reconstruction error, particularly at 12 h. Considering performance across stages and metrics, $\lambda_{\text{temp}}=0.5$ was selected as the default setting.

**Table 2:** Sensitivity analysis of PSTN with respect to the temporal consistency weight $\lambda_{\text{temp}}$ on HVGs = 4000.

HVGs = 4000				
Model	$\lambda_{\text{temp}}$	**0 h (MSE/** $R_{\text{gene}}$ **/** $R_{\text{spot}}$ **)**	**12 h (MSE/** $R_{\text{gene}}$ **/** $R_{\text{spot}}$ **)**	**24 h (MSE/** $R_{\text{gene}}$ **/** $R_{\text{spot}}$ **)**
PSTN	0	0.071/0.979/0.939	0.107/0.966/0.878	0.078/0.978/0.910
PSTN	0.01	0.065/0.980/0.944	0.103/0.966/0.884	0.098/0.979/0.865
PSTN	0.05	0.063/0.981/0.945	0.095/0.971/0.899	0.074/0.979/0.914
PSTN	0.1	0.061/0.981/0.949	0.094/0.972/0.897	0.070/0.980/0.920
**PSTN**	**0.5**	**0.059/0.984/0.956**	**0.089/0.982/0.922**	**0.070/0.977/0.935**
PSTN	1	0.102/0.980/0.926	0.257/0.968/0.785	0.099/0.982/0.875
PSTN	2	0.218/0.976/0.843	0.475/0.955/0.623	0.213/0.983/0.828

Results are reported as MSE/$R_{\text{gene}}$/$R_{\text{spot}}$ at each infection stage. The selected default setting is highlighted in bold.

Across all stages and HVGs settings, PSTN consistently outperformed Tangram, SpaGE, cell2location, and DestVI (Table S1, available as supplementary data at *Bioinformatics* online). At 4000 HVGs, PSTN achieved global MSE values of 0.059, 0.089, and 0.070 at 0 h, 12 h, and 24 h, respectively, whereas all baselines remained near or above 1.0. PSTN also attained markedly higher gene-wise correlations ($R_{\text{gene}}=0.984/0.982/0.977$) and spot-wise correlations ($R_{\text{spot}}=0.956/0.922/0.935$).

Within PSTN, increasing HVGs from 1000 to 4000 monotonically reduced MSE and increased or plateaued both correlation metrics, indicating effective use of additional informative genes. We therefore focus on the 4000-HVGs setting in the main text and report smaller panels in the Supplementary, available as supplementary data at *Bioinformatics* online.

On the independent mouse-cortex dataset, static PSTN and Tangram achieved mean spot-wise cosine similarities of 0.987±0.001 and 0.837 (standard deviation (SD) <0.001), respectively, and 90th-percentile gene-wise cosine similarities of 0.997±0.0003 and 0.975±0.0003 (Table S2, available as supplementary data at *Bioinformatics* online). Because the same genes were used for mapping and evaluation, this experiment assesses static in-panel reconstruction rather than unseen-gene prediction or external validation of temporal regularization.

### 3.4 Dimension-wise reconstruction performance

**Figure 2:**
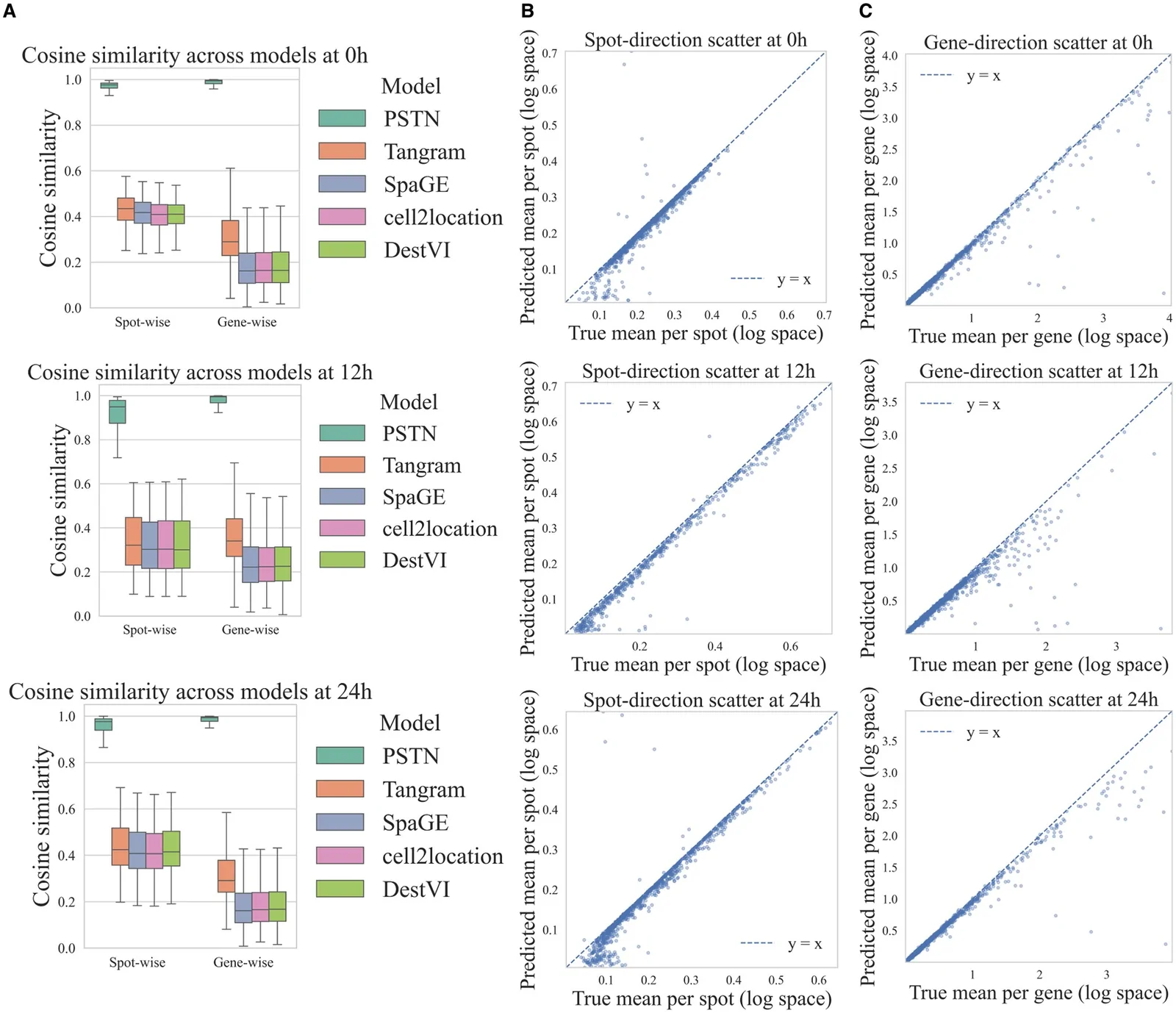
Dimension-wise performance at 4000 HVGs. (A) Gene-wise and spot-wise Pearson correlations for PSTN and baseline methods (Tangram, SpaGE, cell2location, DestVI). (B–C) Predicted versus observed expression at different infection stages in the spot-wise (B) and gene-wise (C) views. Results for additional HVGs settings are shown in Figs S1 and S2, available as supplementary data at *Bioinformatics* online.

As shown in Fig. 2A, PSTN achieves consistently higher gene-wise and spot-wise correlations than all baselines, with narrower distributions and fewer low-end outliers. Tangram performs better than SpaGE, cell2location, and DestVI, particularly for spot-wise correlations, whereas the latter three cluster at comparatively low values. In Fig. 2B and C, predicted and observed profiles align closely along the identity line for both spot-wise and gene-wise views, with no large-scale compression or amplification at higher intensities.

These results indicate that PSTN reduces structured reconstruction errors across both genes and spatial spots, yielding more localized residuals rather than broad gene- or region-level biases.

Gene permutation reduced PSTN $R_{\text{gene}}$ from 0.981–0.985 to 0.734–0.798 and $R_{\text{spot}}$ from 0.919–0.954 to 0.723–0.774, while MSE increased from 0.056–0.092 to 0.308–0.375 (Table S3, available as supplementary data at *Bioinformatics* online). Tangram showed a similar deterioration, supporting that reconstruction depended substantially on valid cross-modal gene correspondence.

### 3.5 PSTN reduces structured residual errors across stages

**Figure 3:**
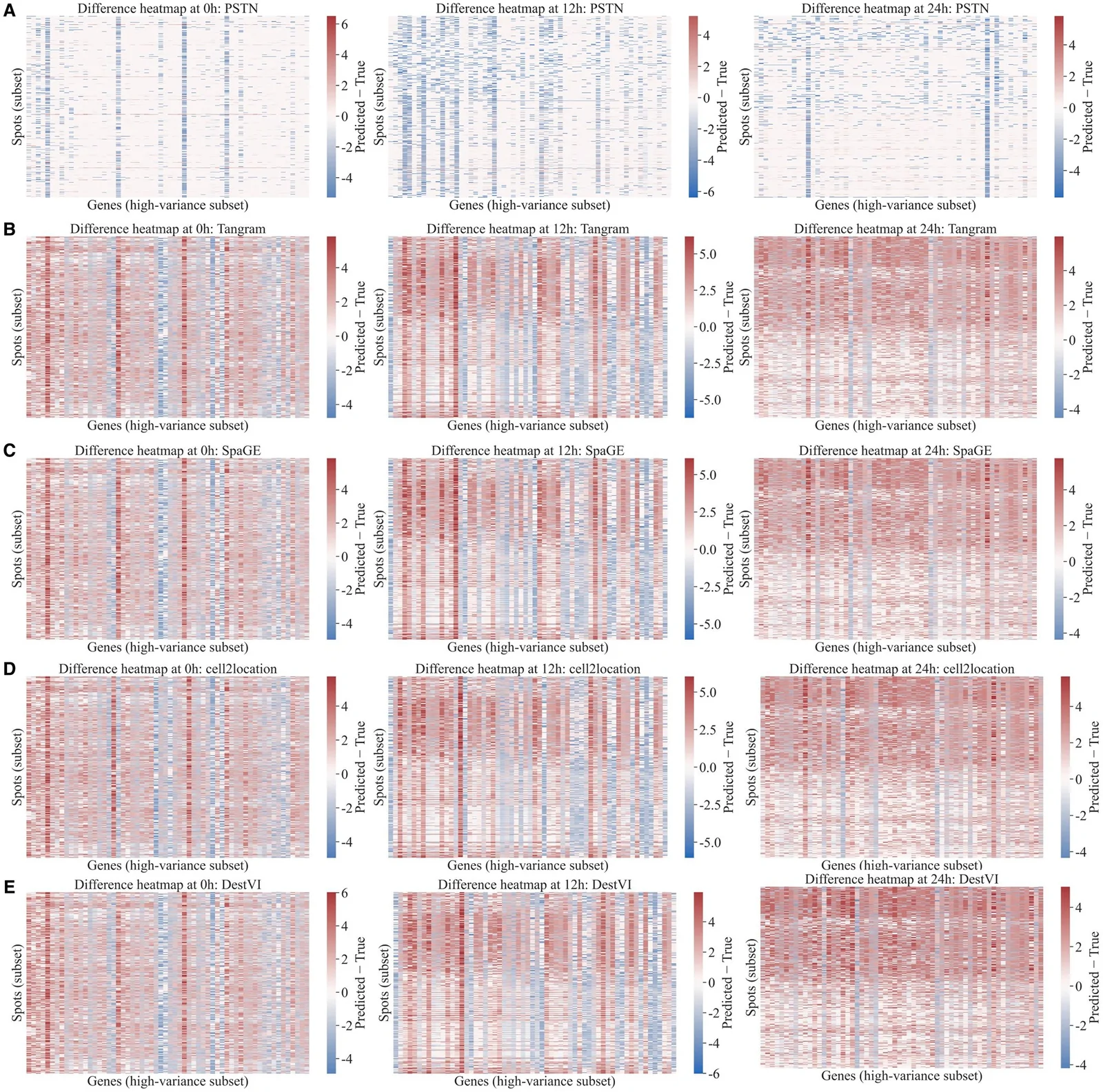
Differential heatmaps (Pred—True) at different time points (0 h, 12 h, and 24 h). Predicted-minus-observed expression at 0 h, 12 h, and 24 h is shown for (A) PSTN, (B) Tangram, (C) SpaGE, (D) cell2location, and (E) DestVI. Columns represent the 60 genes with the highest observed variance, and rows represent spots in their original matrix order, downsampled to ≤500 when necessary. Values are centered at zero (red, overprediction; blue, underprediction). Row order does not indicate spatial adjacency. Additional HVGs settings are shown in Figs S3 and S4, available as supplementary data at *Bioinformatics* online.

Residual maps further showed that PSTN errors were concentrated near zero, with narrow deviations for a limited subset of high-variance genes (Fig. 3A). In contrast, baseline methods displayed broader vertical bands and block-like patterns, indicating stronger gene-level and matrix-structured biases (Fig. 3B–E). Thus, PSTN produced less systematic residual structure across stages.

### 3.6 PSTN recovers cell-type–specific spatial patterns

**Figure 4:**
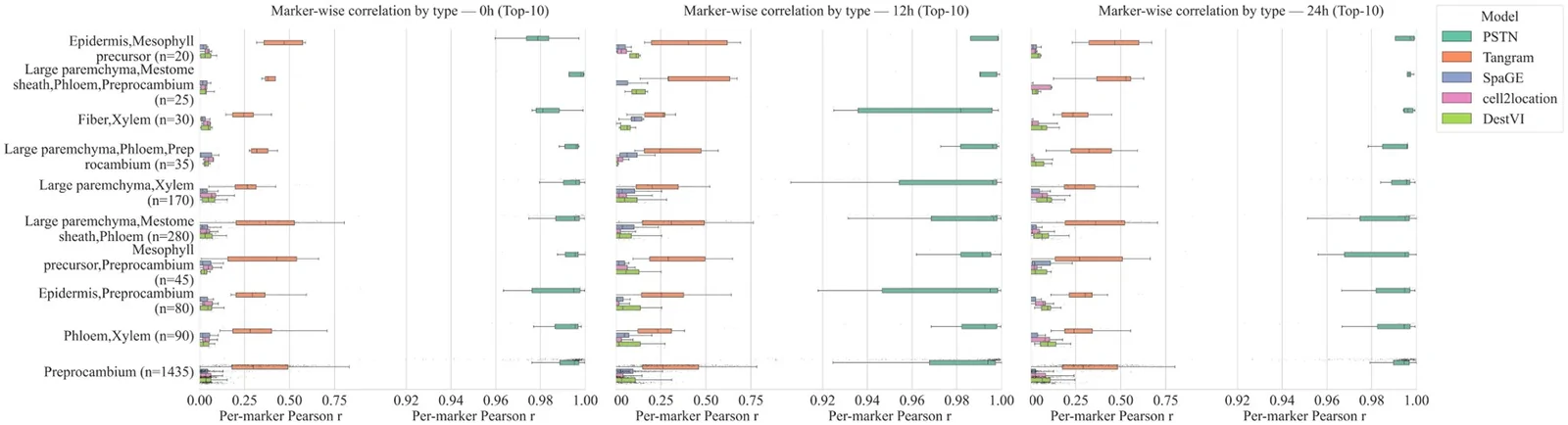
Type-stratified marker-wise correlations across infection time points. Box plots show per-marker Pearson correlations (*r*) between predicted and observed spot-wise expression, stratified by tissue/cell type. Types are the Top 10 ranked by the PSTN median correlation at 24 h; labels include the number of markers used (*n*). Points (semi-transparent) overlay individual markers. PSTN maintains high correlations with narrow dispersion across groups, whereas baseline methods show lower medians and greater variability. Additional HVGs settings are shown in Figs S5 and S6, available as supplementary data at *Bioinformatics* online.

PSTN consistently recovered the spatial expression patterns of tissue/cell-type marker genes across stages (Fig. 4), with median marker-wise correlations generally exceeding 0.95. Tangram showed lower and more dispersed correlations, whereas SpaGE, cell2location, and DestVI performed substantially worse. These results support improved recovery of cell-type-associated marker programs by PSTN.

### 3.7 PSTN learns spatially coherent mapping structures and captures temporal tissue dynamics

Mapping-structure and marker-derived spatial and temporal metrics were calculated as described in Supplementary Methods S1, available as supplementary data at *Bioinformatics* online.

Analysis of the positive mapping weights showed that *M* was broadly distributed but non-uniform (Fig. S7A–D, available as supplementary data at *Bioinformatics* online). Although mapping entropy remained high, the top 5% of reference cells contributed approximately 28%–30% of the positive weight. Neighboring spots also showed greater mapping-vector similarity than random pairs, supporting modest local spatial continuity. Marker-derived dominance remained low, indicating mixed tissue-associated signals within most spots.

PSTN recovered the major temporal changes in tissue-associated marker programs and spatial localization (Fig.S7E–H, available as supplementary data at *Bioinformatics* online). At 12 h, preprocambium-associated signals increased, whereas mesophyll- and mesophyll-precursor-associated signals decreased and partially recovered at 24 h. Moran’s *I* further identified increased mesophyll and preprocambium localization and a transient 12-h peak in mestome-sheath localization. Predicted trends broadly agreed with the observed data, although absolute spatial autocorrelation values differed for some programs. Removing 12 h had only a modest effect on endpoint reconstruction at λ=0.5, whereas excessive temporal regularization impaired performance (Table S4, available as supplementary data at *Bioinformatics* online). Marker-derived weights represent relative tissue-associated programs rather than direct cell-type proportions.

### 3.8 PSTN predictions agree with marker-derived spatial labels

**Figure 5:**
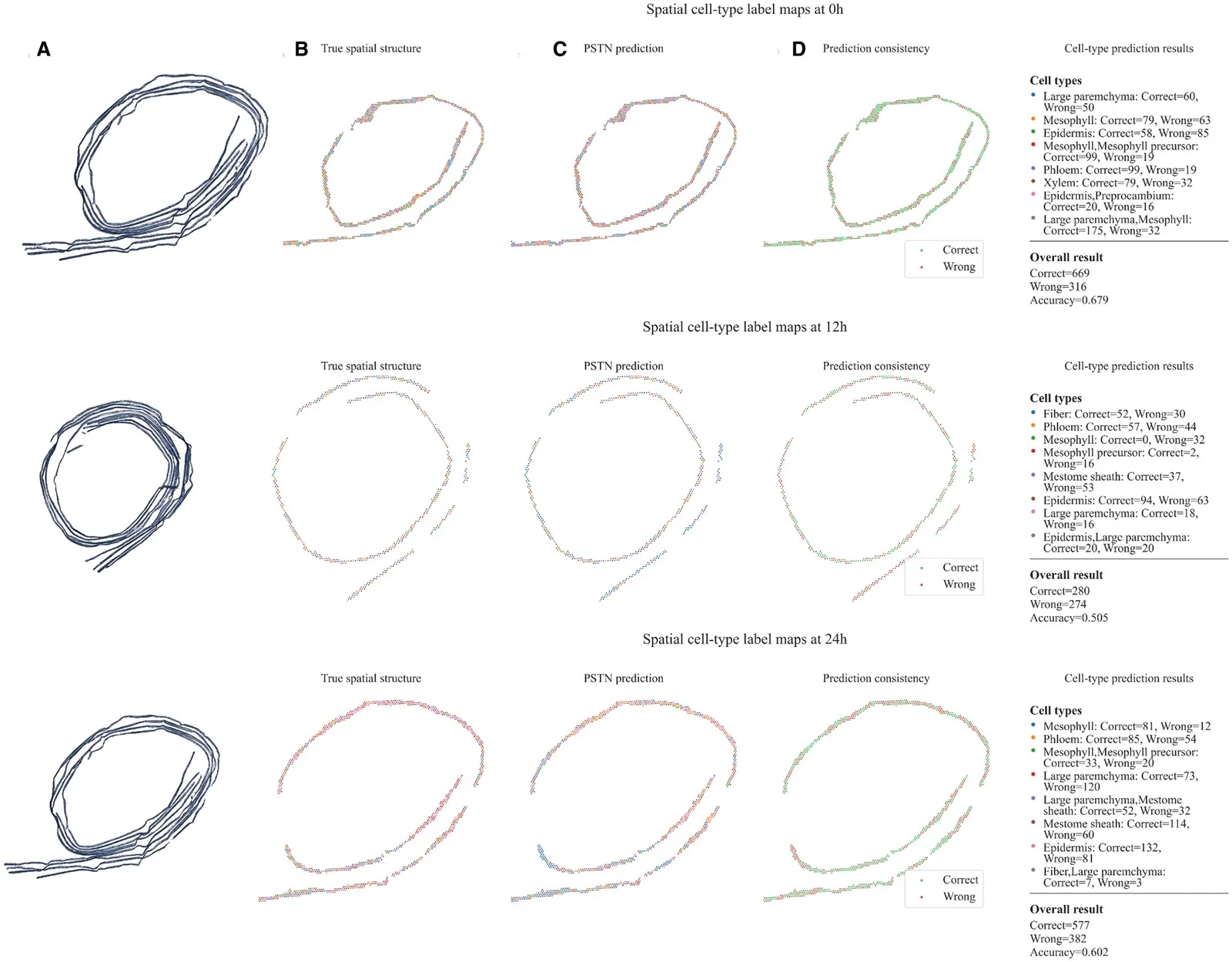
Spatial distributions of marker-derived rice leaf tissue/cell-type labels during *M. oryzae* infection. (A) Rice-leaf histology at 0 h, 12 h, and 24 h. (B–C) Marker-derived reference labels and PSTN predictions. (D) Agreement maps between the two label sets (green, concordant; red, discordant). Recall was calculated as the proportion of evaluable reference-labeled spots correctly matched by PSTN for each tissue/cell type. White spots were excluded because of quality control or insufficient marker support. Additional HVGs settings are shown in Figs S8 and S9, available as supplementary data at *Bioinformatics* online.

PSTN showed strong agreement with marker-derived spatial labels, particularly for epidermis and phloem (Fig. 5). Mesophyll-related recall was lower at 0 h and 12 h but improved at 24 h. Discordant spots were concentrated near tissue boundaries and vascular regions, where histology was also heterogeneous. Recall denotes the proportion of evaluable reference-labeled spots assigned the same label by PSTN.

## 4. Discussion

PSTN extends static cell-to-space mapping by jointly optimizing stage-specific mappings through bidirectional maximum-similarity temporal regularization, without requiring one-to-one spot correspondence. Across stages and HVGs settings, PSTN improved reconstruction and recovered major tissue-associated spatial and temporal patterns. Gene-permutation controls showed that this performance depended substantially on valid cross-modal gene identity. The different magnitudes of degradation observed for PSTN and Tangram likely reflect their distinct mapping parameterizations and optimization constraints. Because gene permutation preserves cell-level covariance, sparsity, and expression totals rather than generating unstructured noise, some residual reconstruction is expected and should not be interpreted as preserved biological correspondence. Accordingly, this control tests dependence on correct gene identity rather than directly comparing perturbation robustness between methods.

Several limitations should be considered. First, *M* is an unconstrained signed mapping matrix; therefore, its raw entries cannot be interpreted as cell probabilities or cell-type proportions. The continuous marker-derived weights used in downstream analyses instead summarize relative tissue-associated transcriptional programs and remain dependent on marker quality and completeness. Second, the temporal loss couples adjacent stages without explicitly encoding the physical time interval. The 12-h spacing reflects the available experimental design rather than a universally optimal sampling interval. Removing the intermediate stage had only a modest effect on endpoint reconstruction, whereas excessive temporal regularization impaired performance, indicating that both sampling frequency and λ should be selected according to biological timescale and empirical validation.

Although the static reconstruction component was additionally evaluated on an independent mouse-cortex scRNA-seq–Visium benchmark, external validation of the temporal component remains limited to the rice–*M. oryzae* system and an early infection window. Additional biological replicates, later infection stages, other cultivars, and matched multi-stage datasets from independent plant or developmental systems will be needed to establish the broader generalizability of the temporal regularization framework. Nevertheless, the independent static benchmark indicates that the underlying reconstruction component remains applicable across organisms and tissue systems, whereas correspondence-free temporal coupling provides a practical strategy for integrating scRNA-seq and ST across dynamic biological stages.

## Supplementary Material

btag637_Supplementary_Data

## Data Availability

The scRNA-seq and spatial transcriptomic datasets analysed in this study are available from the NCBI Sequence Read Archive under BioProject accession numbers PRJNA1121079 and PRJNA1150774. The source code, documentation, and processed inputs required to reproduce the analyses are available at the PSTN GitHub repository. The version used in this study has been archived on Zenodo under DOI: 10.5281/zenodo.21365720.
