# Supplementary material for: Plant Spatio-Temporal Integration Network (PSTN): a framework for dynamic single-cell and spatial transcriptomics in fungal–plant interactions
Source: Bioinformatics. 2026 Aug 22;42(9):btag637. doi: 10.1093/bioinformatics/btag637 (PMC13573595; doi:10.1093/bioinformatics/btag637)
Supplement: Supplementary file 1 [file btag637_supplementary_data.docx]

**Plant Spatio-Temporal Integration Network (PSTN): A framework for dynamic single-cell and spatial transcriptomics in fungal–plant interactions**

**Supplementary data**

Figure S1 Dimension-wise performance at 1,000 HVGs. (A) Box plots of Pearson correlations for gene-wise and spot-wise directions across methods (PSTN, Tangram, SpaGE, cell2location and DestVI). (B) PSTN spot-wise predicted vs. observed scatter (24 h; random/density sampling with a y=x reference). (C) PSTN gene-wise predicted vs. observed scatter (24 h; same plotting protocol as in B). All metrics are computed in the per-spot normalized + log1p space.

Figure S2 Dimension-wise performance at 2,000 HVGs. (A) Box plots of Pearson correlations for gene-wise and spot-wise directions across methods (PSTN, Tangram, SpaGE, cell2location and DestVI). (B) PSTN spot-wise predicted vs. observed scatter (24 h; random/density sampling with a y=x reference). (C) PSTN gene-wise predicted vs. observed scatter (24 h; same plotting protocol as in B). All metrics are computed in the per-spot normalized + log1p space.

Figure S3 Differential heatmaps at 1,000HVGs (Pred − True) at different time (0 h, 12 h and 24 h). (A) PSTN; (B) Tangram; (C) SpaGE; (D) cell2location; (E) DestVI.

Figure S4 Differential heatmaps at 2,000HVGs (Pred − True) at different time (0 h, 12 h and 24 h). (A) PSTN; (B) Tangram; (C) SpaGE; (D) cell2location; (E) DestVI.

Figure S5 Type-stratified marker-wise correlations across infection time points at 1,000 HVGs.

Figure S6 Type-stratified marker-wise correlations across infection time points at 2,000 HVGs.

Figure S7. Mapping-matrix structure and temporal tissue-associated marker dynamics captured by PSTN.

(A) Spot-wise normalized entropy of the positive PSTN mapping weights across 0 h, 12 h, and 24 h. Higher entropy indicates that mapping weights are distributed across a larger number of reference cells. (B) Fraction of the total positive mapping weight contributed by the top 5% of reference cells, reflecting the concentration of the learned mapping. (C) Cosine similarity between mapping vectors of spatially neighboring spots and randomly paired spots. Higher similarity among neighboring spots indicates local spatial continuity of the learned mapping structure. (D) Marker-derived spot dominance, defined as the maximum continuous tissue-associated marker weight within each spot. (E–F) Mean continuous tissue-associated marker weights calculated from the observed and PSTN-reconstructed spatial expression profiles, respectively. (G–H) Moran’s I values of the observed and PSTN-predicted marker-weight distributions, respectively, showing temporal changes in spatial localization. Higher Moran’s I indicates stronger spatial autocorrelation. Marker-derived weights represent relative tissue-associated marker-program activity rather than direct cell-type proportions.

Figure S8. Spatial distribution of rice leaf cell types during *M. oryzae* infection at 1,000HVGs.

Figure S9. Spatial distribution of rice leaf cell types during *M. oryzae* infection at 2,000HVGs.

TABLE S1 Overall performance of PSTN compared with four baselines across different HVG settings

| HVGs1,000 | Model | 0 h(MSE/$R_{\mathrm{gene}}$/$R_{\mathrm{spot}}$) | 12 h(MSE/$R_{\mathrm{gene}}$/$R_{\mathrm{spot}}$) | 24 h(MSE/$R_{\mathrm{gene}}$/$R_{\mathrm{spot}}$) |
| --- | --- | --- | --- | --- |
|  | PSTN | **0.196/0.960/0.942** | **0.257/0.955/0.889** | **0.179/0.966/0.933** |
|  | Tangram | *3.544*/*0.400*/*0.348* | *3.906*/*0.359*/*0.155* | 3.293/*0.357*/*0.436* |
|  | SpaGE | 3.720/0.131/0.288 | 4.061/0.115/0.100 | 3.454/0.111/0.387 |
|  | cell2location | 3.808/0.048/0.271 | 4.126/0.006/0.099 | 3.345/0.045/0.389 |
|  | DestVI | 3.662/0.039/0.289 | 4.191/0.021/0.115 | *3.170*/0.046/0.392 |
| HVGs2,000 | Model | 0 h(MSE/$R_{\mathrm{gene}}$/$R_{\mathrm{spot}}$) | 12 h(MSE/$R_{\mathrm{gene}}$/$R_{\mathrm{spot}}$) | 24 h(MSE/$R_{\mathrm{gene}}$/$R_{\mathrm{spot}}$) |
|  | PSTN | **0.085/0.982/0.961** | **0.146/0.973/0.914** | **0.081/0.983/0.955** |
|  | Tangram | *2.065*/*0.347*/*0.377* | *2.236*/*0.326*/*0.198* | 1.883/*0.320*/*0.428* |
|  | SpaGE | 2.143/0.070/0.333 | 2.314/0.074/0.156 | 1.954/0.045/0.392 |
|  | cell2location | 2.224/0.052/0.317 | 2.368/0.017/0.151 | 1.916/0.045/0.392 |
|  | DestVI | 2.202/0.026/0.322 | 2.459/0.008/0.011 | *1.789*/0.069/0.402 |
| HVGs4,000 | Model | 0 h(MSE/$R_{\mathrm{gene}}$/$R_{\mathrm{spot}}$) | 12 h(MSE/$R_{\mathrm{gene}}$/$R_{\mathrm{spot}}$) | 24 h(MSE/$R_{\mathrm{gene}}$/$R_{\mathrm{spot}}$) |
|  | PSTN | **0.059/0.984/0.956** | **0.089/0.982/0.922** | **0.070/0.977/0.935** |
|  | Tangram | *1.077*/*0.328*/*0.383* | *1.200*/*0.302*/*0.232* | 0.986/*0.315*/*0.410* |
|  | SpaGE | 1.096/0.037/0.359 | 1.217/0.046/0.210 | 1.005/0.018/0.388 |
|  | cell2location | 1.133/0.039/0.349 | 1.265/0.007/0.209 | 1.002/0.021/0.384 |
|  | DestVI | 1.119/0.030/0.351 | 1.296/0.031/0.209 | *0.964*/0.044/0.395 |

Table S2. Cosine similarity–based comparison of static PSTN and Tangram on an independent mouse cortex scRNA-seq–Visium dataset

| Method | Mean spot-wise cosine | 90th-percentile gene-wise cosine |
| --- | --- | --- |
| Static PSTN | 0.987 ± 0.001 | 0.997 ± 0.0003 |
| Tangram | 0.837 ± <0.001 | 0.975 ± 0.0003 |

Table S3. Reconstruction performance of PSTN using real versus gene-permuted scRNA-seq inputs

| Models | MSE | $R_{\mathrm{gene}}$ | $R_{\mathrm{spot}}$ |
| --- | --- | --- | --- |
| Real_PSTN_0H | 0.059 | 0.985 | 0.954 |
| Real_PSTN_12H | 0.092 | 0.981 | 0.919 |
| Real_PSTN_24H | 0.056 | 0.985 | 0.944 |
| Gene_permuted_PSTN_0H | 0.308 | 0.798 | 0.723 |
| Gene_permuted_PSTN_12H | 0.337 | 0.734 | 0.774 |
| Gene_permuted_PSTN_24H | 0.375 | 0.788 | 0.743 |
| Real_Tangram_0H | 1.165 | 0.348 | 0.402 |
| Real_Tangram_12H | 1.314 | 0.388 | 0.225 |
| Real_Tangram_24H | 1.258 | 0.363 | 0.428 |
| Gene_permuted_Tangram_0H | 3.552 | 0.137 | 0.035 |
| Gene_permuted_Tangram_12H | 3.058 | 0.174 | 0.037 |
| Gene_permuted_Tangram_24H | 3.452 | 0.143 | 0.036 |

Note: The real-input and gene-permuted models were obtained from a separate negative-control rerun under identical settings; therefore, the real-input values are not identical to those reported in Table 2.

Table S4. Reconstruction performance of PSTN using only the 0 h and 24 h snapshots under different temporal regularization strengths

| Temporal setting | λ | Time point | MSE | $R_{\mathrm{gene}}$ | $R_{\mathrm{spot}}$ |
| --- | --- | --- | --- | --- | --- |
| 0 h–24 h only | 0 | 0 h | 0.068 | 0.978 | 0.939 |
| 0 h–24 h only | 0 | 24 h | 0.079 | 0.978 | 0.908 |
| 0 h–24 h only | 0.5 | 0 h | 0.064 | 0.980 | 0.951 |
| 0 h–24 h only | 0.5 | 24 h | 0.073 | 0.973 | 0.932 |
| 0 h–24 h only | 2 | 0 h | 0.247 | 0.965 | 0.821 |
| 0 h–24 h only | 2 | 24 h | 0.207 | 0.983 | 0.831 |

**Supplementary Methods S1. Mapping-structure and marker-derived spatial and temporal analyses**

For direct analysis of the mapping matrix $M$, positive weights were normalized within each spatial spot. Normalized mapping entropy was calculated as $H/log(N)$, where $N$is the number of reference cells, and top-5% concentration was defined as the summed weight of the largest 5% of cell contributions. Spatial neighbors were defined using a six-nearest-neighbor graph based on spot coordinates, and cosine similarities between neighboring $M$columns were compared with randomly sampled spot pairs. Positive weights were used for the primary analysis, with absolute-weight summaries used as sensitivity checks.

Marker-based analyses retained single-type marker sets containing at least 10 genes in the 4,000-HVG panel. Predicted spatial expression was calculated as $M^{\top}A_{\mathrm{raw}}$, clipped at zero, normalized to ${10}^{4}$counts per spot, and log1p-transformed. Marker genes were standardized using the pooled observed ST profiles across all stages, averaged within each marker set, and converted to continuous tissue-associated weights by row-wise softmax. Spot dominance was defined as the maximum marker-derived weight. Mean marker weights and Moran’s $I$ were calculated for predicted and observed profiles at each stage using the same six-nearest-neighbor spatial graph.

**Supplementary Note S2. Baseline implementation and harmonized evaluation**

Tangram, the SpaGE-style method, cell2location, and DestVI were applied independently to the 0 h, 12 h, and 24 h datasets. For each HVG setting, genes shared across the corresponding scRNA-seq and spatial transcriptomic datasets were retained and arranged in a common order. Raw expression matrices were retrieved preferentially from AnnData.layers["raw_X"], followed by a dimensionally compatible AnnData.raw.X, and otherwise from AnnData.X.

For Tangram, the scRNA-seq and spatial matrices were independently normalized to ${10}^{4}$counts per cell or spot and log1p-transformed before model fitting. Tangram was run in cell-level mapping mode for 2,000 epochs, with other optimization parameters left at the defaults of the installed Tangram-sc version. The output mapping matrix was aligned to the original cell and spot identifiers and converted to a cell-by-spot matrix for subsequent expression reconstruction.

The SpaGE-style baseline was fitted using normalized log1p expression. Genes with zero variance in either modality were removed, and the cell and spot feature spaces were separately reduced by truncated singular-value decomposition to at most 512 dimensions. The reduced representations were standardized and aligned by canonical correlation analysis using up to 30 components, a maximum of 1,000 iterations, and a tolerance of ${10}^{-6}$. Cosine similarities between the resulting cell and spot embeddings were converted to spot-to-cell weights using a row-wise softmax with temperature 0.07.

For cell2location, cell-type reference signatures were calculated as the mean raw expression of each available cell type. When curated cell-type annotations were unavailable, pseudo-cell types were generated by Leiden clustering at resolution 0.8 after normalization, log1p transformation, principal-component analysis, and construction of a 15-nearest-neighbor graph using 30 principal components. cell2location was trained for 2,000 epochs using N_cells_per_location = 5 and detection_alpha = 20, and posterior cell-type abundance was estimated using 1,000 posterior samples. Non-integer spatial input values were rounded to non-negative integers before model fitting when required. The resulting spot-by-cell-type abundance was converted to a cell-by-spot mapping matrix by distributing each type-specific abundance equally among the corresponding reference cells.

For DestVI, the same available or Leiden-derived cell-type labels were used. A CondSCVI reference model was first trained on the scRNA-seq data for 800 epochs. DestVI was then initialized from the fitted CondSCVI model and trained on the corresponding spatial dataset for 2,000 epochs. The inferred spot-by-cell-type proportions were converted to a cell-by-spot mapping matrix by distributing each type-specific proportion equally among reference cells assigned to that type.

For all four baselines, reconstructed raw spatial expression was obtained as:

$$\hat{B}_{\mathrm{raw}}=M^{\top}A_{\mathrm{raw}},$$

where $M$denotes the cell-by-spot mapping matrix and $A_{\mathrm{raw}}$denotes the raw scRNA-seq count matrix. Negative reconstructed values were clipped to zero when applicable. Predicted and observed spatial matrices were then normalized separately to ${10}^{4}$counts per spot and log1p-transformed. All methods were evaluated in this common expression space using global MSE, mean gene-wise Pearson correlation across spots, and mean spot-wise Pearson correlation across genes. Exact scripts and package versions are provided in the accompanying archived code repository.
